# 

**Published:** 1875-03

**Authors:** 


CONTENTS.

| | PAGE |
|---|---|
| Active Oxygen | 69 |
| Feeding the Sick | 72 |
| The Perfect Man | 73 |
| Modes of Dying | 75 |
| How We Made Bread | 77 |
| The Most Deadly Disease | 83 |
| Richmond Hill | 84 |
| Mrs. Lyman's Letter | 86 |
| The House of Steinway | 87 |
| Inquiries Answered | 88 |
| Pneumonia and Steam-Coils | 89 |
| Vinegar and Flesh | 90 |
| Spring Medicine | 91 |
| Immortality of the Eye | 93 |
| Eating Wood | 94 |
| Deformed Feet | 95 |
| Decaying Teeth | 96 |
| How Bones Heal | 100 |

